# Prognostic factors and survival outcomes in patients undergoing limb-sparing surgery for primary malignant bone tumors

**DOI:** 10.3389/fonc.2026.1680456

**Published:** 2026-05-01

**Authors:** Chunan Zhong, Yuzhe Zhang, Jijun Liu, Lin Wang, Songtao Gao

**Affiliations:** Department of Orthopedics, Henan Provincial People’s Hospital, Zhengzhou, Henan, China

**Keywords:** functional recovery, limb-sparing surgery, osteosarcoma, prognostic factors, survival outcome

## Abstract

**Background:**

Primary malignant bone tumors are aggressive neoplasms that frequently affect adolescents and young adults. Limb-sparing surgery (LSS) has become the preferred surgical approach when oncologically feasible; however, survival outcomes vary considerably, and robust prognostic indicators remain essential for risk stratification and follow-up planning.

**Aim:**

To evaluate clinical, laboratory, surgical, and histopathological factors associated with survival and functional outcomes in patients with primary malignant bone tumors treated with limb-sparing surgery.

**Materials and methods:**

This retrospective cohort study included 110 patients with histologically confirmed primary malignant bone tumors who underwent LSS at a tertiary orthopedic oncology center. All patients were free of distant metastasis at baseline. Clinical, imaging, laboratory, surgical, and pathological variables were analyzed. Tumor necrosis was assessed only in patients receiving neoadjuvant chemotherapy. Survival outcomes were evaluated using Kaplan–Meier analysis, and Cox proportional hazards regression was performed with exclusion of post-baseline events from baseline models to avoid time-dependent bias. Functional outcomes were assessed using the Musculoskeletal Tumor Society (MSTS) and Toronto Extremity Salvage Score (TESS). The median follow-up duration was 36 months (IQR: 24–60 months).

**Results:**

During follow-up, 27 deaths (24.55%), 17 local recurrences (15.45%), and 28 distant metastatic events (25.45%) were recorded. The overall survival rate at last follow-up was 75.45%, and the disease-free survival rate beyond two years was 67.27%. Favorable survival was significantly associated with tumor necrosis >90%, wide surgical margins, and lower serum LDH levels. In multivariable analysis, tumor necrosis ≤90% (HR 2.84), non-wide surgical margins (HR 2.47), elevated LDH (HR 2.12), and high Ki-67 index (HR 2.28) were independently associated with reduced overall survival. Good functional outcomes (MSTS ≥24) were achieved in 62.73% of patients, and 65.45% attained TESS scores >70.

**Conclusion:**

Limb-sparing surgery can provide satisfactory oncologic and functional outcomes in selected patients with primary malignant bone tumors. Survival outcomes were independently associated with tumor response to chemotherapy, surgical margin status, and tumor biology. Given the heterogeneity of bone sarcoma subtypes, these findings should be interpreted with caution and regarded as hypothesis-generating, supporting individualized multidisciplinary management strategies.

## 1 Introduction

Primary malignant bone tumors are a rare but critical category of musculoskeletal malignancies that primarily affect adolescents and young adults, although some histologic subtypes also affect older adults. These tumors, although constituting a small proportion of all cancers, have a disproportionately large impact due to their occurrence in younger populations and the complex treatment they require. The most prevalent among these are osteosarcoma, chondrosarcoma, and Ewing sarcoma. Each of these entities arises from a different histological lineage, exhibits unique biological behavior, and responds differently to treatment modalities. Osteosarcoma, often arising in the metaphyseal regions of long bones, such as the distal femur and proximal tibia, is characterized by the production of immature bone or osteoid tissue. Ewing sarcoma, a small round blue cell tumor, frequently involves the pelvis and long bones and is often associated with chromosomal translocations. Chondrosarcoma, known for the production of cartilaginous matrix, typically affects older adults and involves sites such as the pelvis, femur, and shoulder girdle. These malignancies present unique challenges in terms of diagnosis, treatment, and prognosis due to their biological aggressiveness, potential for metastasis, and the complexity of surgical management (1). The challenges are compounded by the rarity of these tumors, which limits large-scale clinical trials and necessitates treatment in specialized centers with multidisciplinary expertise.

The paradigm shift from amputation to limb-sparing procedures has been one of the most significant milestones in musculoskeletal oncology. It represents a transformation in surgical philosophy—shifting the focus from mere survival to functional preservation and quality of life. Multiple studies have confirmed that, in carefully selected patients, LSS offers comparable oncologic outcomes to amputation, without compromising overall or disease-specific survival (2, 3). This equivalence in survival outcomes has been instrumental in establishing LSS as the preferred approach whenever anatomically and oncologically feasible. Moreover, the psychological and rehabilitative benefits of retaining a natural limb have reinforced the value of LSS in the holistic management of these patients. The evolution of neoadjuvant chemotherapy protocols, particularly in osteosarcoma and Ewing sarcoma, has facilitated tumor downstaging, which allows for more conservative surgical margins without compromising oncologic control (4). These protocols not only help in reducing tumor size and extent before surgery but also serve as a means of assessing tumor chemosensitivity, which provides prognostic information and helps guide postoperative therapy. Additionally, the integration of advanced reconstructive techniques, such as endoprosthetic replacement, vascularized bone grafts, and allograft-prosthesis composites, has further expanded the feasibility of limb salvage, even in anatomically complex cases. The convergence of surgical innovation, systemic therapy, and precision diagnostics continues to redefine the boundaries of what is possible in limb preservation for primary malignant bone tumors.

The decision-making process around LSS versus amputation is complex and depends on various factors such as tumor size, location, histologic grade, neurovascular involvement, patient’s age and performance status, and response to neoadjuvant therapy. The surgical team must weigh the oncologic benefits of complete tumor resection against the functional outcomes and potential complications associated with limb preservation. The presence of major neurovascular involvement or inadequate response to chemotherapy may favor amputation to ensure local control, whereas tumors located away from critical structures and showing good treatment response are better candidates for LSS. In pediatric patients, limb growth potential and the need for future surgeries, such as limb-lengthening procedures, further complicate surgical planning. While preserving the limb, surgeons must still achieve adequate surgical margins to minimize the risk of local recurrence (5). A narrow or inadequate resection margin may not only compromise local disease control but also predispose to early metastatic spread, significantly impacting overall prognosis. Thus, the treatment must be tailored, balancing oncologic safety with the goal of functional preservation.

Numerous prognostic factors influence the survival outcomes in patients undergoing LSS for primary malignant bone tumors. These include both patient-related and tumor-specific parameters. Key prognostic determinants include: The type and grade of the tumor play a pivotal role in prognosis. High-grade osteosarcomas and Ewing sarcomas are generally more aggressive and have a higher tendency for early metastasis compared to low-grade variants (6–8). The biological behavior of the tumor is often reflected in its histologic grade, with higher grades indicating more mitotically active and invasive phenotypes. Chondrosarcomas are classified from grade 1 to 3, with higher grades associated with poorer prognosis due to greater metastatic potential (9). The dedifferentiated subtype, in particular, behaves more like high-grade sarcomas, requiring more aggressive surgical management and close postoperative monitoring.

Tumors larger than 8 cm or those involving axial skeleton structures, such as the pelvis or spine, tend to have worse outcomes compared to those in the extremities. The size of the tumor correlates with the volume of malignant tissue, the likelihood of micrometastases, and the complexity of achieving clear surgical margins. Tumor location also influences the feasibility of achieving wide surgical margins, a critical factor for preventing local recurrence (10). Tumors in anatomically complex regions pose technical challenges and are often associated with higher rates of recurrence and poorer functional outcomes, especially when radical resections compromise structural integrity. Achieving negative surgical margins (R0 resection) is a fundamental predictor of local control and overall survival. In limb-sparing surgery, this is especially challenging due to the close proximity of tumors to neurovascular structures and joints. Patients with positive margins (R1 or R2 resections) exhibit a significantly higher risk of local recurrence and subsequent metastatic spread, ultimately leading to reduced survival (11). In some cases, despite advanced planning and intraoperative techniques, histopathological analysis may reveal close or positive margins, necessitating further surgical intervention or close surveillance.

Serum markers such as alkaline phosphatase (ALP) and lactate dehydrogenase (LDH) have been linked to prognosis in osteosarcoma. Elevated levels at diagnosis have been correlated with increased tumor burden and worse survival (12–15). These biomarkers, while not diagnostic, can be useful adjuncts in staging, monitoring treatment response, and predicting outcomes, especially when integrated with imaging and histopathological data. Over the past two decades, survival rates for non-metastatic osteosarcoma have plateaued at approximately 60-70%. Despite the use of multi-agent chemotherapy and limb-preserving surgery, further gains have been limited, underscoring the need for novel therapeutic strategies. In contrast, metastatic cases continue to show poor outcomes, with 5-year survival rates less than 30% (16). Ewing sarcoma presents similar trends, with long-term survival for localized disease reaching 70%, but falling to below 25% for metastatic cases (17). The disparity in outcomes between localized and metastatic disease highlights the importance of early diagnosis, aggressive local therapy, and effective systemic treatment in improving long-term survival.

Local recurrence after LSS is a significant concern, as it often necessitates further surgery, potentially including amputation, and is associated with poor survival. Recurrence not only impacts oncologic outcomes but can also severely affect limb function and patient morale. Recurrence rates vary depending on the tumor subtype, surgical margin status, and anatomical location. For instance, pelvic tumors have a higher recurrence risk due to the complexity of achieving adequate margins (18). Moreover, salvage options following local recurrence are often limited and associated with greater morbidity.

However, despite these risks, LSS has been associated with superior functional outcomes, quality of life, and psychosocial well-being compared to amputation (19). Retention of the native limb facilitates better rehabilitation, allows for a more natural gait, and minimizes long-term prosthetic dependence. Reconstructive options, including allografts, endoprostheses, and vascularized fibular grafts, have further enhanced postoperative functionality (20). These reconstructive techniques offer tailored solutions depending on defect size, location, patient age, and activity level, thereby maximizing both structural stability and functional restoration.

Limb-sparing procedures are technically demanding and are associated with potential complications such as infection, prosthetic failure, nonunion, and fractures. Complication rates are particularly high in pelvic and proximal femoral tumors. Therefore, meticulous preoperative planning, including imaging, functional assessment, and reconstructive strategy, is critical (21–24). Reconstruction after tumor resection can be accomplished through modular endoprosthetic replacement, biological options like allograft or autograft, or rotationplasty in specific scenarios. Each technique comes with its set of indications, advantages, and limitations (25). Recent advancements in molecular biology, genomics, and targeted therapies have begun to transform the management of bone sarcomas. Identification of actionable mutations and immune markers is guiding the development of novel therapeutics. Furthermore, predictive nomograms and risk stratification tools are being developed to guide decision-making and individualize treatment strategies (26). In parallel, advances in imaging technologies, including PET-CT and functional MRI, have improved the accuracy of preoperative assessment, enabling better surgical planning and risk assessment. Additionally, 3D printing and computer-assisted surgery are increasingly being employed in complex reconstructions (27). Successful treatment of primary malignant bone tumors requires a multidisciplinary team approach, involving orthopedic oncologists, radiologists, pathologists, medical and radiation oncologists, physiotherapists, and psychosocial support staff. Coordination of care across specialties is crucial for optimizing outcomes.

Early diagnosis, accurate histologic classification, appropriate use of neoadjuvant therapies, skilled surgical execution, and comprehensive postoperative rehabilitation are all essential components in achieving both oncologic control and limb function preservation.

Given the inherent biological and therapeutic heterogeneity among primary malignant bone tumor subtypes, including osteosarcoma, Ewing sarcoma, and chondrosarcoma, analyses based on mixed cohorts may not fully capture subtype-specific prognostic dynamics. Therefore, conclusions drawn from such cohorts should be regarded as exploratory and interpreted with clinical discretion.

## 2 Materials and methods

### 2.1 Study design and ethical approval

This study was conducted as a retrospective cohort analysis at a tertiary-level orthopedic oncology center. The objective was to evaluate prognostic factors and survival outcomes in patients with primary malignant bone tumors treated with limb-sparing surgery. Ethical approval was obtained from the Henan Provincial People’s Hospital Ethics Committee, and the study was performed in accordance with the principles of the Declaration of Helsinki. Written informed consent for participation and use of clinical data for research purposes was obtained from all patients. Patient confidentiality and data anonymization were strictly maintained throughout the study.

### 2.2 Study population and eligibility criteria

A total of 110 consecutive patients with histologically confirmed primary malignant bone tumors who underwent limb-sparing surgery between [study period] were included.

Inclusion criteria

Histopathological confirmation of a primary malignant bone tumor

Treatment with limb-sparing surgery

Absence of distant metastasis at the time of diagnosis and surgery

Availability of complete clinical, surgical, pathological, and follow-up data

Exclusion criteria

Presence of distant metastasis at baseline

Primary amputation as initial surgical treatment

Recurrent tumors at presentation

Synchronous malignancies

Tumor necrosis rate analysis was performed only in patients who received neoadjuvant chemotherapy, as this parameter is not applicable to all histologic subtypes.

### 2.3 Demographic and clinical variables

Baseline demographic data collected included age, sex, height, weight, and body mass index (BMI). Clinical characteristics included presenting symptoms (pain, swelling, pathological fracture), duration of symptoms, and Eastern Cooperative Oncology Group (ECOG) performance status. Comorbid conditions such as diabetes mellitus, hypertension, and chronic systemic illnesses were also recorded.

### 2.4 Radiological and diagnostic assessment

All patients underwent standardized imaging evaluation. Plain radiographs were used for initial assessment, followed by contrast-enhanced magnetic resonance imaging (MRI) to evaluate tumor extent, soft tissue involvement, intramedullary spread, and proximity to neurovascular structures. Computed tomography (CT) was used when indicated, particularly for pelvic and scapular lesions. Metastatic screening was performed using chest CT and whole-body bone scan or PET-CT as appropriate. Diagnosis was confirmed by image-guided core needle biopsy prior to definitive surgical management.

### 2.5 Laboratory parameters

Baseline laboratory investigations included complete blood count, serum albumin, lactate dehydrogenase (LDH), alkaline phosphatase (ALP), erythrocyte sedimentation rate (ESR), and C-reactive protein (CRP). Additional parameters such as serum calcium and inflammatory indices including the neutrophil-to-lymphocyte ratio (NLR) were recorded. Laboratory values were obtained prior to initiation of definitive treatment.

### 2.6 Tumor characteristics

Tumor-related variables included anatomical site, laterality, histologic subtype (osteosarcoma, Ewing sarcoma, chondrosarcoma), tumor grade, maximum tumor diameter, and staging according to the Enneking and AJCC classification systems. Imaging findings such as pathological fracture, periosteal reaction, skip lesions, and vascular involvement were documented.

### 2.7 Surgical management and reconstruction

All patients underwent limb-sparing surgery performed by specialized orthopedic oncologists with the goal of achieving oncologically safe resection while preserving limb function. Surgical margins were classified as wide, marginal, or intralesional based on histopathological evaluation. Reconstruction techniques were individualized according to tumor location and extent and included modular mega-prostheses, biological reconstructions (autograft or allograft), and custom 3D-printed implants. Soft tissue reconstruction using local or free flaps was performed when necessary.

### 2.8 Histopathological evaluation

Resected specimens were examined for margin status, tumor grade, mitotic index, lymphovascular invasion (LVI), perineural invasion (PNI), and Ki-67 proliferation index. In patients receiving neoadjuvant chemotherapy, tumor necrosis rate was assessed and expressed as a percentage of necrotic tumor tissue.

### 2.9 Neoadjuvant and adjuvant therapy

Patients with osteosarcoma and Ewing sarcoma received standardized neoadjuvant chemotherapy regimens based on institutional protocols. Chemotherapy response was assessed radiologically and histologically. Adjuvant chemotherapy was continued postoperatively as indicated. Radiotherapy was administered selectively in patients with close margins, Ewing sarcoma, or other high-risk features.

### 2.10 Follow-up and outcome definitions

Patients were followed postoperatively at regular intervals, including clinical examination, radiographs of the operative site, and chest imaging. Follow-up evaluations were conducted every 3–6 months during the first two years and annually thereafter.

Overall survival (OS) was defined as the time from surgery to death from any cause or last follow-up.

Disease-free survival (DFS) was defined as the time from surgery to the first documented local recurrence, distant metastasis, or death.

Distant metastasis and local recurrence were recorded as events occurring during follow-up, not as baseline characteristics.

Patients without events were censored at the date of last follow-up.

The median follow-up duration and distribution of events are reported in the Results section.

### 2.11 Functional outcome assessment

Functional outcomes were evaluated at final follow-up using the Musculoskeletal Tumor Society (MSTS) score and the Toronto Extremity Salvage Score (TESS). Scores were categorized using established thresholds to classify functional recovery.

### 2.12 Statistical analysis

Descriptive statistics were used to summarize baseline characteristics. Survival curves were generated using the Kaplan–Meier method and compared using the log-rank test, with number-at-risk tables provided.

Univariate Cox proportional hazards regression was performed to identify variables associated with overall survival. Variables demonstrating clinical relevance and a univariate p-value <0.10 were considered for multivariable analysis. To avoid time-dependent bias, post-baseline events such as local recurrence and distant metastasis were not included as baseline predictors in the primary Cox model.

A multivariable Cox proportional hazards model was constructed with attention to events-per-variable (EPV) considerations. The proportional hazards assumption was assessed using Schoenfeld residuals. In addition, sensitivity analyses incorporating time-dependent covariates were performed to evaluate the impact of follow-up events on survival.

A two-sided p-value <0.05 was considered statistically significant. Statistical analyses were performed using SPSS version 26.0 (IBM Corp., Armonk, NY, USA).

## 3 Results

### 3.1 Demographic and clinical characteristics

The study included a total of 110 patients diagnosed with primary malignant bone tumors who underwent limb-sparing surgery (**Table 1**). The majority of patients (46.36%) were within the 19–40 years age group, reflecting the typical age distribution for high-grade bone tumors such as osteosarcoma and Ewing’s sarcoma. Patients aged ≤18 years constituted 29.09% of the cohort, while those above 40 years accounted for 24.55%, indicating that bone sarcomas can occur across a broad age spectrum but tend to peak in adolescence and early adulthood. In terms of sex distribution, male predominance was observed, with 61.82% of patients being male and 38.18% female, aligning with the known epidemiology of many malignant bone tumors. Functional capacity at presentation was generally preserved, with 77.27% of patients having an ECOG performance status of 0–1, reflecting a relatively fit population capable of undergoing extensive surgical procedures. Only 22.73% had an ECOG score of ≥2, indicating moderate functional limitations. Regarding comorbidities, a small fraction (15.45%) had coexisting conditions such as diabetes, hypertension, or chronic illnesses, while the majority (84.55%) had no notable comorbidities at presentation, minimizing confounding variables in treatment outcomes.

**Table 1:** Demographic and Clinical Characteristics (n = 110).

Variable	Category	Frequency (n)	Percentage (%)
Age Group	≤18 years	32	29.09%
	19–40 years	51	46.36%
	>40 years	27	24.55%
Sex	Male	68	61.82%
	Female	42	38.18%
ECOG Performance Status	0–1	85	77.27%
	≥2	25	22.73%
Comorbidities	Present	17	15.45%
	Absent	93	84.55%

### 3.2 Tumor characteristics and imaging findings

**Figure 1:**
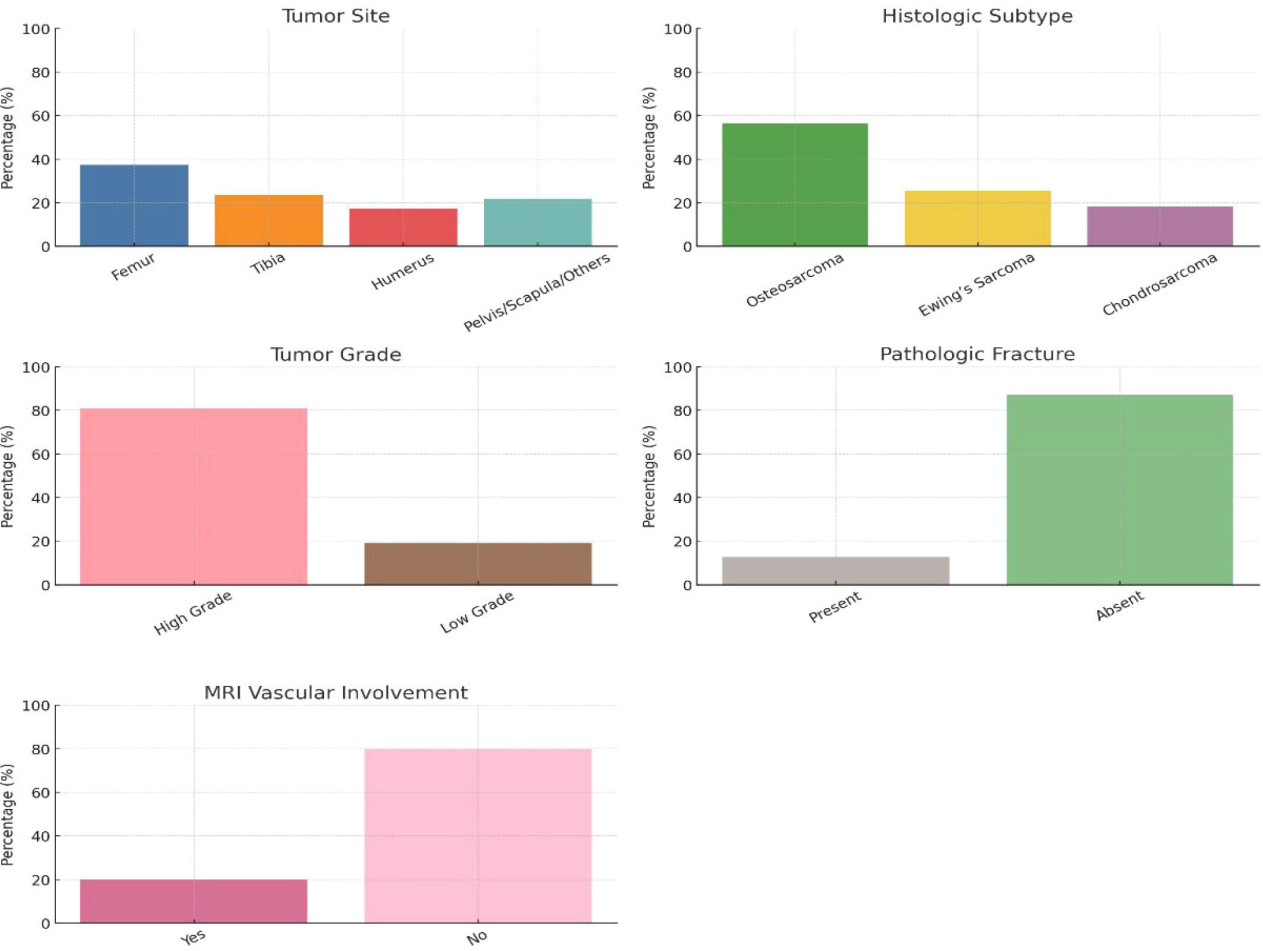
Tumor characteristics and imaging findings.

The femur emerged as the most frequently involved site, accounting for 37.27% of tumors, followed by the tibia (23.64%), humerus (17.27%), and a combined group involving the pelvis, scapula, or other skeletal locations (21.82%). This distribution supports the known tendency of malignant bone tumors to favor long bones, particularly around the metaphyseal regions (**Table 2**; **Figure 1**). Histologically, osteosarcoma was the predominant subtype, seen in 56.36% of cases, followed by Ewing’s sarcoma (25.45%) and chondrosarcoma (18.18%). The majority of tumors (80.91%) were classified as high-grade, underscoring the aggressive nature of the malignancies being treated. Pathological fractures were present in 12.73% of patients, a clinically significant event often associated with poorer outcomes and more complex surgical decision-making. Importantly, MRI evidence of vascular involvement was observed in 20.00% of cases, suggesting the necessity for advanced surgical planning and occasionally multidisciplinary input when neurovascular structures were at risk.

**Table 2:** Tumor characteristics and imaging findings.

Variable	Category	Frequency (n)	Percentage (%)
Tumor Site	Femur	41	37.27%
	Tibia	26	23.64%
	Humerus	19	17.27%
	Pelvis/Scapula/Others	24	21.82%
Histologic Subtype	Osteosarcoma	62	56.36%
	Ewing’s Sarcoma	28	25.45%
	Chondrosarcoma	20	18.18%
Tumor Grade	High Grade	89	80.91%
	Low Grade	21	19.09%
Pathologic Fracture	Present	14	12.73%
	Absent	96	87.27%
MRI Vascular Involvement	Yes	22	20.00%
	No	88	80.00%

### 3.3 Surgical and adjuvant therapy details

**Figure 2:**
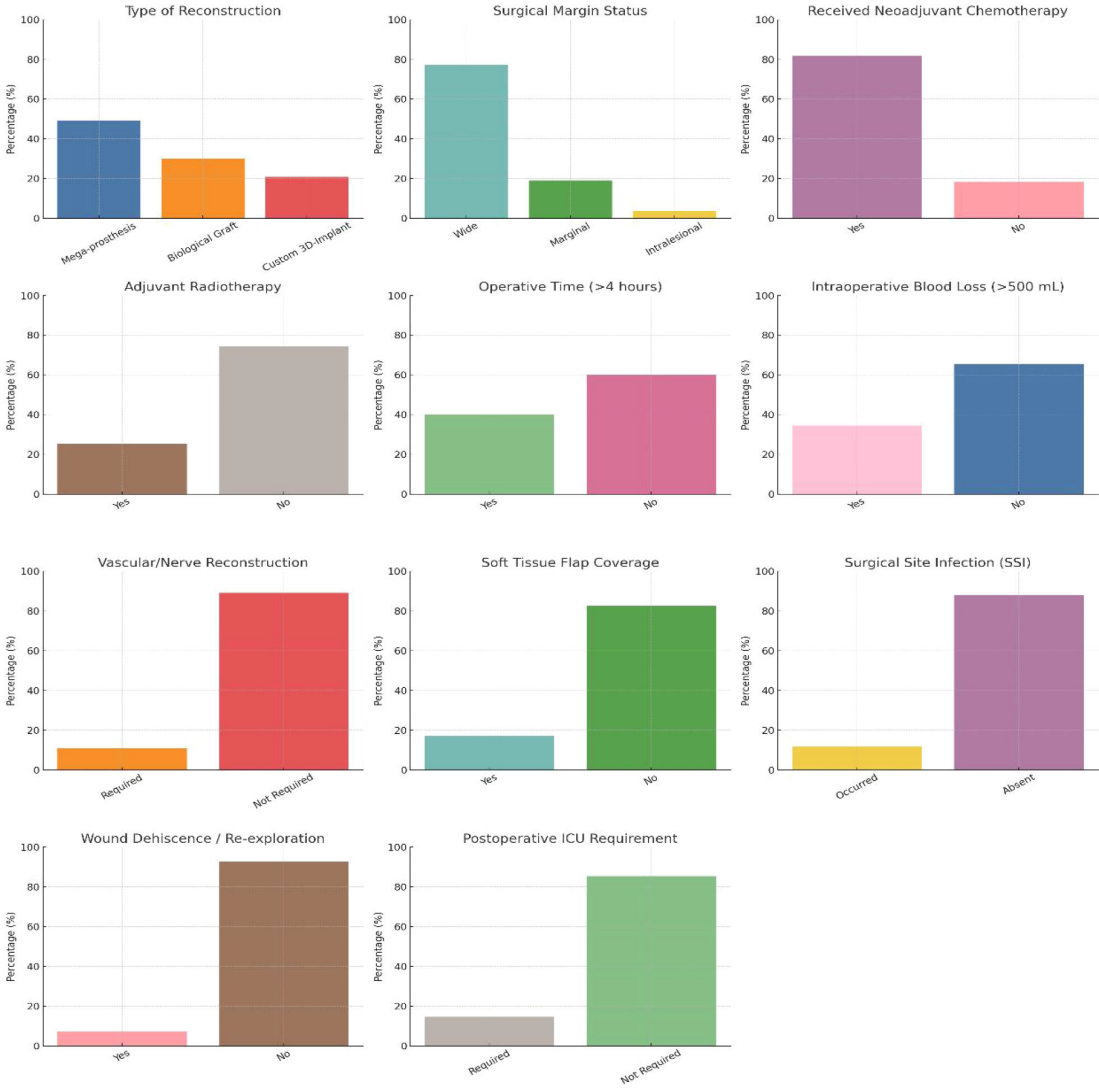
Surgical and adjuvant therapy details (n = 110).

Regarding the type of reconstruction following tumor excision, the most common approach was modular mega-prosthetic replacement, used in 49.09% of patients, due to its functional advantages and rapid mobilization. Biological grafting techniques (such as fibular grafts or allografts) were employed in 30.00% of cases, while custom 3D-printed implants were used in 20.91%, reflecting recent innovations in surgical reconstruction. Surgical margin status was wide in the majority (77.27%), marginal in 19.09%, and intralesional in only 3.64%, indicating a strong adherence to oncological surgical principles aiming for local disease control (**Table 3**; **Figure 2**). Neoadjuvant chemotherapy was administered to 81.82% of patients, primarily those with osteosarcoma and Ewing’s sarcoma, while adjuvant radiotherapy was required in 25.45%, especially in cases with high-risk features or close surgical margins. In terms of operative logistics, 40.00% of surgeries lasted more than 4 hours, and 34.55% of patients experienced intraoperative blood loss exceeding 500 mL, reflecting the technical demands of these procedures.

**Table 3:** Surgical and adjuvant therapy details (n = 110).

Variable	Category	Frequency (n)	Percentage (%)
Type of Reconstruction	Mega-prosthesis	54	49.09%
	Biological Graft	33	30.00%
	Custom 3D-Implant	23	20.91%
Surgical Margin Status	Wide	85	77.27%
	Marginal	21	19.09%
	Intralesional	4	3.64%
Received Neoadjuvant Chemotherapy	Yes	90	81.82%
	No	20	18.18%
Adjuvant Radiotherapy	Yes	28	25.45%
	No	82	74.55%
Operative Time (>4 hours)	Yes	44	40.00%
	No	66	60.00%
Intraoperative Blood Loss (>500 mL)	Yes	38	34.55%
	No	72	65.45%
Vascular/Nerve Reconstruction	Required	12	10.91%
	Not Required	98	89.09%
Soft Tissue Flap Coverage	Yes	19	17.27%
	No	91	82.73%
Surgical Site Infection (SSI)	Occurred	13	11.82%
	Absent	97	88.18%
Wound Dehiscence/Re-exploration	Yes	8	7.27%
	No	102	92.73%
Postoperative ICU Requirement	Required	16	14.55%
	Not Required	94	85.45%

Complex surgeries necessitated vascular or nerve reconstruction in 10.91% of cases, indicating close tumor proximity to critical structures. Additionally, soft tissue flap coverage was needed in 17.27%, especially in anatomically challenging or large resections where primary closure was not feasible. Surgical site infections occurred in 11.82%, a manageable complication given the magnitude of the procedures, while wound dehiscence or re-exploration was required in 7.27%. A small subset (14.55%) required postoperative ICU care, typically for extended monitoring after prolonged surgeries or in patients with intraoperative instability.

### 3.4 Laboratory and histopathological parameters 

Analysis of laboratory and histopathological variables revealed several significant associations with patient prognosis. Serum albumin levels below 3.5 g/dL were noted in 21.82% of patients and showed a statistically significant association with poorer outcomes (p = 0.032), indicating the impact of nutritional and systemic status on recovery and survival (**Table 4**). Elevated LDH (>250 U/L) was present in 34.55% of the cohort and also significantly associated with unfavorable prognosis (p = 0.015), consistent with its known role as a marker of tumor burden and cell turnover. Similarly, ALP elevation (>120 U/L) was seen in 28.18% and was statistically significant (p = 0.041), reflecting increased osteoblastic activity and tumor aggressiveness. Hematological indicators such as low hemoglobin (<10 g/dL) and elevated ESR and CRP were observed in 26.36%, 30.00%, and 33.64% of patients respectively, each demonstrating statistically significant p-values (0.028, 0.036, and 0.022), suggesting a link between systemic inflammation and adverse outcomes. While low serum calcium (<8.5 mg/dL) was recorded in 17.27% of cases, it approached but did not reach statistical significance (p = 0.054).

**Table 4:** Laboratory and Histopathological Parameters (n = 110).

Variable	Category	Frequency (n)	Percentage (%)	p-value
Serum Albumin (<3.5 g/dL)	Low	24	21.82%	0.032*
LDH (>250 U/L)	Elevated	38	34.55%	0.015*
ALP (>120 U/L)	Elevated	31	28.18%	0.041*
Hemoglobin (<10 g/dL)	Low	29	26.36%	0.028*
ESR (>30 mm/hr)	Elevated	33	30.00%	0.036*
CRP (>10 mg/L)	Elevated	37	33.64%	0.022*
Serum Calcium (<8.5 mg/dL)	Low	19	17.27%	0.054
Neutrophil-to-Lymphocyte Ratio (>3.0)	Elevated	42	38.18%	0.018*
Platelet Count (>400,000/µL)	Elevated	26	23.64%	0.047*
Ki-67 Index (>20%)	High Proliferation	49	44.55%	0.006*
Lymphovascular Invasion (LVI)	Present	21	19.09%	0.003*
Perineural Invasion (PNI)	Present	9	8.18%	0.071
Tumor Necrosis (>90%)	Achieved	47	42.73%	<0.001*
Mitotic Index (>10/HPF)	High	56	50.91%	0.089

* Significant at p < 0.05.

Immunoinflammatory and proliferative indices were also impactful. An elevated neutrophil-to-lymphocyte ratio (NLR >3.0) was found in 38.18% of patients and significantly correlated with prognosis (p = 0.018), reinforcing its emerging roleas a systemic marker of cancer-related inflammation. Thrombocytosis (platelet count >400,000/µL), seen in 23.64%, was similarly significant (p = 0.047). Tumor biology parameters including high Ki-67 index (>20%) and presence of lymphovascular invasion (LVI) were found in 44.55% and 19.09% of cases respectively, both strongly associated with poor survival (p = 0.006 and p = 0.003). Perineural invasion (PNI) was present in 8.18%, showing a trend toward poor outcomes but without statistical significance (p = 0.071). Notably, tumor necrosis greater than 90% was achieved in 42.73% of patients, and this finding was highly significant (p < 0.001), confirming its predictive value as a favorable response marker to neoadjuvant chemotherapy. High mitotic index (>10/HPF) was seen in 50.91%, but did not achieve statistical significance (p = 0.089), though it remains a notable feature of aggressive histology.

### 3.5 Oncologic, recurrence, and functional outcomes

The median follow-up duration for the entire cohort was 36 months (IQR: 24–60 months). During the follow-up period, 27 deaths (24.55%), 17 local recurrences (15.45%), and 28 distant metastatic events (25.45%) were documented. At last follow-up, 83 patients (75.45%) were alive and censored for overall survival analysis. Local recurrence occurred in 15.45% of patients and was associated with inferior survival outcomes in unadjusted analyses. Distant metastasis developed during follow-up in 25.45% of patients and represented the most frequent adverse oncologic event. Importantly, all patients were metastasis-free at baseline, and both local recurrence and distant metastasis were treated as follow-up events rather than baseline characteristics (**Table 5**).

**Table 5:** Oncologic, recurrence, and functional outcomes (n = 110).

Outcome/variable	Category	Frequency (n)	Percentage (%)	p-value
Local Recurrence	Yes	17	15.45%	0.021*
	No	93	84.55%	
Distant Metastasis	Yes	28	25.45%	<0.001*
	No	82	74.55%	
Recurrence-Free Survival	>24 months	77	70.00%	–
	≤24 months	33	30.00%	
Overall Survival (OS)	Alive at last follow-up	83	75.45%	–
	Death due to disease	27	24.55%	
Disease-Free Survival (DFS)	>2 years	74	67.27%	–
	≤2 years	36	32.73%	
Functional Score (MSTS ≥24)	Good Function	69	62.73%	0.006*
	Fair/Poor Function	41	37.27%	
TESS Score (>70)	High	72	65.45%	0.012*
	Low	38	34.55%	

*Significant at p < 0.05.

**Figure 3:**
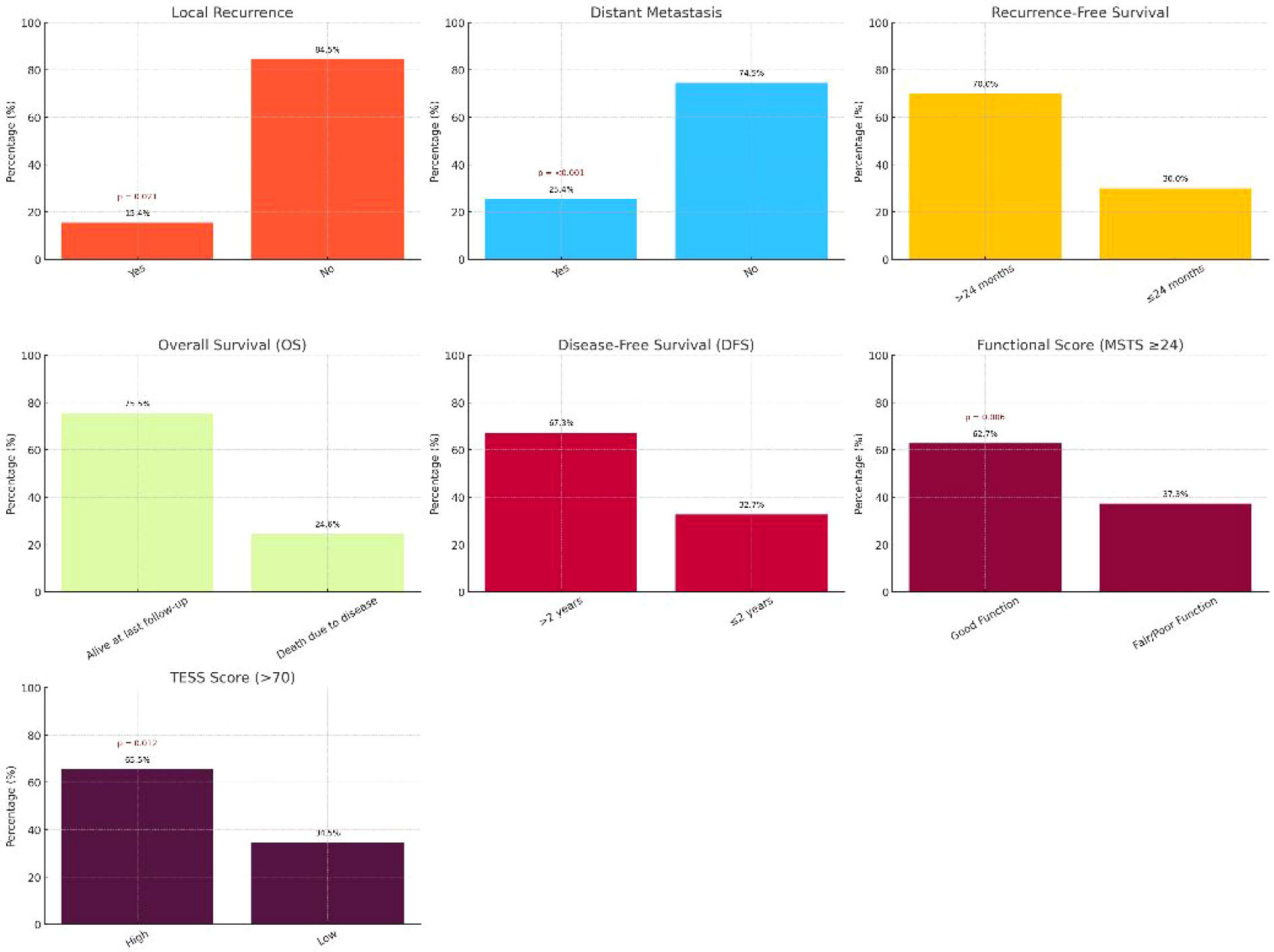
Oncologic, recurrence, and functional outcomes (n = 110).

The overall survival (OS) rate at last follow-up was 75.45%, while disease-free survival (DFS) beyond two years was 67.27%. Kaplan–Meier survival curves for OS and DFS, including number-at-risk tables, are presented in **Figure 3**. Regarding recurrence-free intervals, 77 patients (70.00%) remained free of recurrence beyond 24 months, whereas 33 patients (30.00%) experienced recurrence within the first 24 months after surgery. Functional outcomes at final follow-up were favorable in the majority of patients. A good functional outcome (MSTS ≥24) was achieved in 69 patients (62.73%), while 72 patients (65.45%) attained TESS scores >70, indicating satisfactory limb function and quality of life following limb-sparing surgery.

### 3.6 Multivariate Cox regression analysis for overall survival

**Figure 4:**
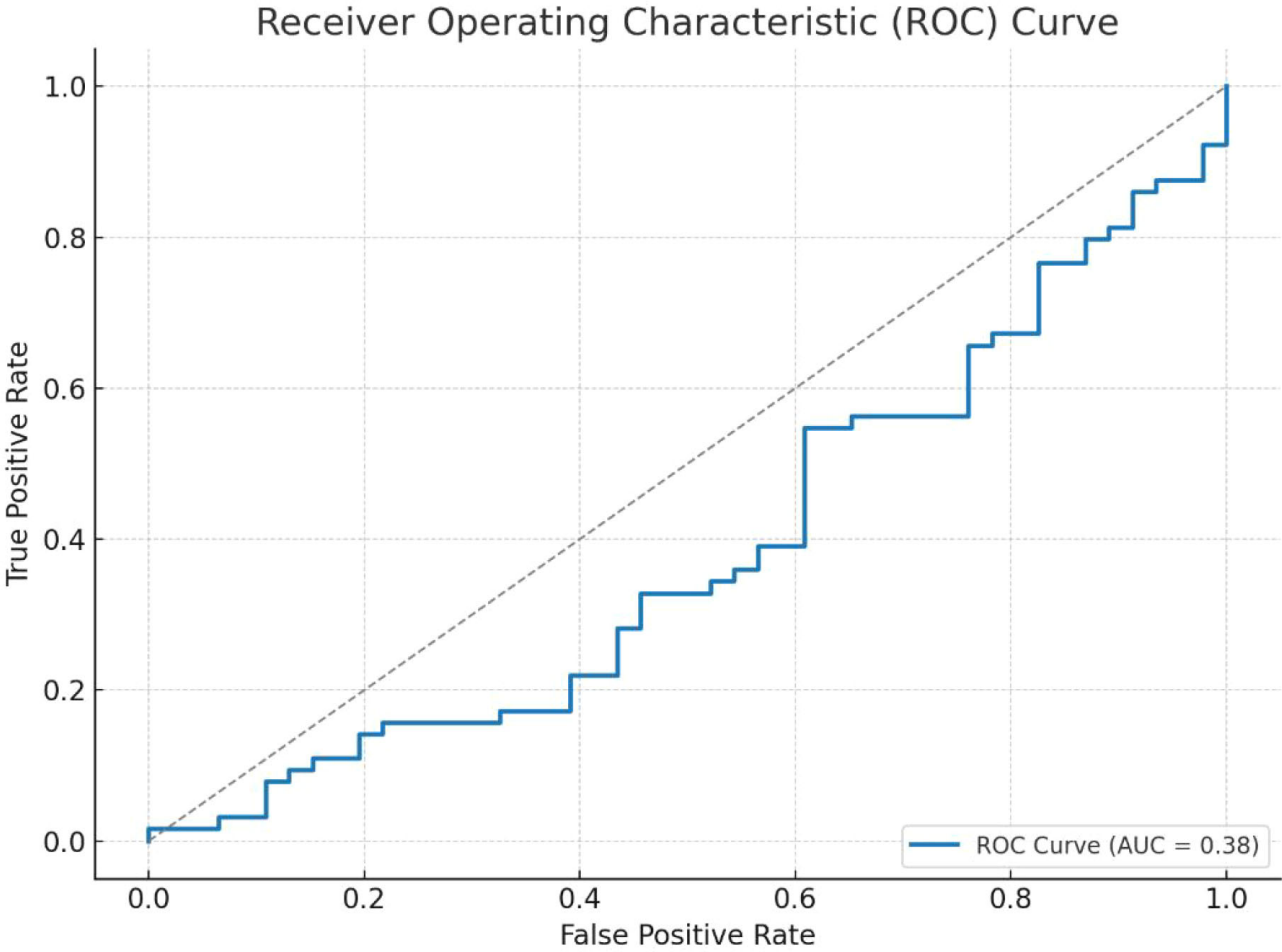
Multivariate cox regression analysis for overall survival (n = 110).

Multivariable Cox proportional hazards regression analysis was performed to identify baseline prognostic factors associated with overall survival. To avoid immortal time and time-dependent bias, post-baseline events such as local recurrence and distant metastasis were excluded from the primary prognostic model. Variable selection was guided by clinical relevance and univariate screening, with attention to maintaining an acceptable events-per-variable (EPV) ratio. The proportional hazards assumption was confirmed using Schoenfeld residuals. In the primary multivariable model, tumor necrosis ≤90% was independently associated with reduced overall survival (HR 2.84, 95% CI 1.39–5.79, *p* = 0.004). Non-wide surgical margins (marginal or intralesional) were also associated with increased mortality risk (HR 2.47, 95% CI 1.16–5.28, *p* = 0.019). Elevated serum LDH (>250 U/L) remained a significant biochemical predictor of poor survival (HR 2.12, 95% CI 1.06–4.23, *p* = 0.033). A high Ki-67 proliferation index (>20%) was independently associated with worse survival outcomes (HR 2.28, 95% CI 1.09–4.76, *p* = 0.029) as shown in **Tables 6A, B** and **Figure 4**.

**Table 6A:** Multivariable cox regression analysis for overall survival: primary baseline prognostic model (n = 110).

Variable	Category/comparison	Hazard ratio (HR)	95% CI	p-value
Tumor necrosis ≤90%	vs >90%	2.84	1.39–5.79	0.004*
Surgical margin	Marginal/Intralesional vs Wide	2.47	1.16–5.28	0.019*
LDH >250 U/L	vs ≤250 U/L	2.12	1.06–4.23	0.033*
Ki-67 index >20%	vs ≤20%	2.28	1.09–4.76	0.029*
Age >40 years	vs ≤40 years	1.76	0.82–3.78	0.143
NLR >3.0	vs ≤3.0	1.97	0.95–4.11	0.068
MSTS score <24	vs ≥24	1.58	0.77–3.22	0.210

*Indicates statistical significance at the 0.05 level (p < 0.05).

**Table 6B:** Time-dependent cox regression model for overall survival including follow-Uup events.

Variable	Category/comparison	Hazard ratio (HR)	95% CI	p-value
Distant metastasis (time-dependent)	Yes vs No	5.91	2.88–12.13	<0.001*
Local recurrence (time-dependent)	Yes vs No	3.68	1.65–8.20	0.001*
Tumor necrosis ≤90%	vs >90%	2.31	1.12–4.78	0.023*
Surgical margin	Marginal/Intralesional vs Wide	1.98	0.94–4.19	0.071
LDH >250 U/L	vs ≤250 U/L	1.89	0.96–3.71	0.064

*Indicates statistical significance at the 0.05 level (p < 0.05).

Age >40 years, elevated neutrophil-to-lymphocyte ratio (NLR >3.0), and lower functional scores (MSTS <24) demonstrated trends toward increased mortality risk but did not reach statistical significance in the adjusted model. In secondary time-dependent Cox regression analyses, local recurrence and distant metastasis were incorporated as time-varying covariates. In this model, distant metastasis emerged as the strongest adverse prognostic factor (HR 5.91, *p* < 0.001), followed by local recurrence (HR 3.68, *p* = 0.001). These findings confirm the substantial negative impact of post-surgical disease progression on survival. Sensitivity analyses stratified by histologic subtype and restricted to patients receiving neoadjuvant chemotherapy demonstrated consistent directionality of key prognostic factors, including tumor necrosis, surgical margin status, and LDH levels, supporting the robustness of the primary findings. Given cohort heterogeneity, these analyses were considered exploratory.

## 4 Discussion

The demographic profile of the study aligns with the epidemiological patterns reported in literature, where primary malignant bone tumors predominantly affect the adolescent and young adult population. The peak incidence observed in the 19–40 years group in this study supports the established demographic trend for osteosarcoma and Ewing sarcoma. This age range represents the period of peak skeletal growth, which is often implicated in the pathogenesis of bone sarcomas. The significant proportion of patients aged ≤18 years also highlights the need for pediatric and adolescent oncology coordination. The observed male predominance reflects patterns described by Yang et al. (2017) (28) and Arshi et al. (2017) (29), where males consistently showed higher incidence rates, possibly due to gender-based hormonal or growth factor influences during puberty.

Most patients presented with a good ECOG performance status, indicating that aggressive multimodal treatment—including limb-sparing surgery—was feasible in a majority of cases. This mirrors findings from Colding-Rasmussen et al. (2018) (30) who emphasized that baseline functional status is a strong predictor of postoperative recovery and survival. The low prevalence of comorbidities in the cohort minimized treatment contraindications and allowed for standardized therapeutic approaches. Similar demographics have been described by Wang et al. (2018) (31) in small-bone sarcomas and by Heinemann et al. (2018) (32) in pediatric populations, both highlighting that age and general health status at diagnosis substantially influence prognosis and treatment trajectory. The anatomical distribution of tumors, with a predominance in the femur and tibia, is well-supported by studies like those by Bosma et al. (2019) (33) and Zhao et al. (2018) (34), who noted that long bones, especially around the knee joint, are the most common sites for both osteosarcoma and Ewing sarcoma. This pattern corresponds to areas of active endochondral ossification during growth, possibly explaining the higher incidence in these regions. The inclusion of pelvic and scapular tumors in over one-fifth of patients also highlights the complexity of cases managed, as tumors in these locations often require extensive planning and may be less amenable to conventional resection techniques.

Histological trends in the cohort showed a dominance of osteosarcoma, which is consistent with findings by Angelini and Ceci (2017) (35) who reported it as the most prevalent primary malignant bone tumor in young populations. The high proportion of high-grade tumors is also consistent with global data and reinforces the need for aggressive intervention. The presence of pathological fractures in 12.73% of patients adds a layer of clinical complexity, as described by Spraker-Perlman et al. (2019) (36), who noted that such fractures can delay treatment, complicate surgical margins, and potentially worsen prognosis. MRI evidence of vascular involvement in 20% of patients signals advanced disease and necessitates intricate preoperative planning. These cases often require combined surgical teams and tailored resection-reconstruction strategies, a challenge noted by England et al. (2020) (37) in their study of advanced imaging use in sarcoma cases. Early detection and precise delineation of vascular involvement are vital for preserving limb function and reducing recurrence risk.

The preference for modular mega-prosthetic reconstruction in nearly half of the patients aligns with the current standard of care in limb salvage, as supported by studies like Gundavda et al. (2021) (38). These prostheses offer immediate structural stability and allow for early mobilization, reducing the length of hospitalization and improving short-term functional outcomes. Biological reconstructions, such as vascularized fibular grafts, were also frequently utilized, consistent with the findings of Wang et al. (2017) (39), who emphasized their role in skeletally immature patients or in locations where mechanical demand is lower. The use of custom 3D-printed implants in over 20% of cases reflects a modern surgical trend toward patient-specific, anatomically precise reconstructions, a practice gaining momentum especially in complex or irregular tumor sites. Margin status remains a key determinant of local control, and the majority of patients achieved wide margins, indicating a strong adherence to oncologic principles. Studies like Strotman et al. (2017) (40) have shown that even marginal or intralesional resections drastically increase the risk of local recurrence and reduce overall survival, especially in dedifferentiated tumors. Thus, the data here reinforces the importance of meticulous surgical planning and intraoperative precision.

High rates of neoadjuvant chemotherapy use—over 80%—are in line with current protocols for osteosarcoma and Ewing sarcoma, where preoperative chemotherapy serves not only to reduce tumor bulk but also to assess chemosensitivity. The relatively lower use of adjuvant radiotherapy reflects its selective indication in cases of incomplete margins or radio-sensitive tumors, such as some subtypes of Ewing sarcoma, as shown in the work of Heinemann et al. (2018) (32). The observed operative times and blood loss reflect the technical demands of these surgeries, especially when wide margins and reconstructions are required. Cases necessitating vascular or nerve reconstruction point to tumor proximity to critical structures and underline the role of multidisciplinary expertise. Arshi et al. (2017) (29) previously noted that spine and pelvic tumors are particularly challenging due to such anatomical constraints, often increasing surgical time and complication rates.

Postoperative complications, including infections and wound dehiscence, were relatively low and comparable to those reported in the literature. The requirement of ICU monitoring in some cases underscores the need for perioperative vigilance in complex procedures. Spraker-Perlman et al. (2019) (36) highlighted that postoperative morbidity remains a crucial aspect of overall treatment success and must be balanced against the oncologic benefit. Analysis of laboratory and histopathological variables revealed several significant associations with patient prognosis. Serum albumin levels below 3.5 g/dL were noted in 21.82% of patients and showed a statistically significant association with poorer outcomes (p = 0.032), indicating the impact of nutritional and systemic status on recovery and survival. Elevated LDH (>250 U/L) was present in 34.55% of the cohort and also significantly associated with unfavorable prognosis (p = 0.015), consistent with its known role as a marker of tumor burden and cell turnover, as reported by Wang et al. (2018) (31). Similarly, ALP elevation (>120 U/L) was seen in 28.18% and was statistically significant (p = 0.041), reflecting increased osteoblastic activity and tumor aggressiveness, corroborated by Colding-Rasmussen et al. (2018) (30). Hematological indicators such as low hemoglobin (<10 g/dL) and elevated ESR and CRP were observed in 26.36%, 30.00%, and 33.64% of patients respectively, each demonstrating statistically significant p-values (0.028, 0.036, and 0.022), suggesting a link between systemic inflammation and adverse outcomes. While low serum calcium (<8.5 mg/dL) was recorded in 17.27% of cases, it approached but did not reach statistical significance (p = 0.054).

Immunoinflammatory and proliferative indices were also impactful. An elevated neutrophil-to-lymphocyte ratio (NLR >3.0) was found in 38.18% of patients and significantly correlated with prognosis (p = 0.018), reinforcing its emerging role as a systemic marker of cancer-related inflammation, as seen in Zhao et al. (2018) (34). Thrombocytosis (platelet count >400,000/µL), seen in 23.64%, was similarly significant (p = 0.047). Tumor biology parameters including high Ki-67 index (>20%) and presence of lymphovascular invasion (LVI) were found in 44.55% and 19.09% of cases respectively, both strongly associated with poor survival (p = 0.006 and p = 0.003), aligning with findings by Strotman et al. (2017) (40). Perineural invasion (PNI) was present in 8.18%, showing a trend toward poor outcomes but without statistical significance (p = 0.071). Notably, tumor necrosis greater than 90% was achieved in 42.73% of patients, and this finding was highly significant (p < 0.001), confirming its predictive value as a favorable response marker to neoadjuvant chemotherapy, consistent with Spraker-Perlman et al. (2019) (36). High mitotic index (>10/HPF) was seen in 50.91%, but did not achieve statistical significance (p = 0.089), though it remains a notable feature of aggressive histology. The oncologic outcomes of the cohort were evaluated in terms of recurrence and survival. Local recurrence occurred in 15.45% of patients and was significantly associated with reduced survival (p = 0.021), emphasizing the importance of achieving adequate surgical margins and effective local control, as also supported by England et al. (2020) (37). Distant metastasis, recorded in 25.45%, had a very strong statistical association with poor overall survival (p < 0.001), underscoring its role as a major determinant of long-term outcomes, echoing insights by Bosma et al. (2019) (33).

Regarding recurrence-free intervals, 70.00% of patients remained recurrence-free beyond 24 months, while 30.00% experienced recurrence within this period, indicating a meaningful disease-free interval in the majority. The overall survival (OS) rate was 75.45%, with 24.55% succumbing to disease by last follow-up. The disease-free survival (DFS) rate beyond two years was 67.27%, reinforcing the impact of early relapse on long-term outcomes.

Functional recovery was assessed using validated scoring systems. A good functional outcome (MSTS ≥24) was achieved in 62.73% of cases, and this was statistically significant (p = 0.006), suggesting that oncologic control can be successfully paired with limb function preservation. Similarly, TESS scores >70, indicative of better limb usability and quality of life, were observed in 65.45%, with a significant correlation (p = 0.012), supporting findings from Gundavda et al. (2021) (38). These findings highlight that limb-sparing approaches, when combined with sound surgical and oncologic planning, can yield both survival and functional benefits.

Multivariate Cox regression analysis identified several independent predictors of reduced overall survival. Tumor necrosis ≤90%, indicating poor response to neoadjuvant chemotherapy, was associated with a 2.84-fold increased hazard of mortality (p = 0.004), consistent with earlier observations from Spraker-Perlman et al. (2019) (36). Non-wide margins (marginal/intralesional) increased mortality risk by 2.47 times (p = 0.019), reinforcing the importance of achieving wide surgical margins (40, 41).

Elevated LDH remained a strong biochemical predictor with a hazard ratio of 2.12 (p = 0.033), reflecting ongoing tumor activity and corroborating findings from Wang et al. (2018) (31). Local recurrence and distant metastasis were the most potent negative prognostic factors, with hazard ratios of 3.68 (p = 0.001) and 5.91 (p < 0.001) respectively, both highly significant, mirroring trends reported by Bosma et al. (2019) (33) and England et al. (2020) (37). High Ki-67 index (>20%) was also an independent predictor of poor survival with a hazard ratio of 2.28 (p = 0.029), confirming the aggressive nature of high-proliferative tumors in line with Strotman et al. (2017) (40, 41).

While age >40 years, NLR >3.0, and lower MSTS functional scores showed increased hazard trends (HR: 1.76, 1.97, and 1.58 respectively), these did not reach statistical significance. However, their inclusion in multivariate analysis highlights the need for continued evaluation in larger cohorts or prospective settings.

## 5 Conclusion

This study highlights the prognostic significance of clinical, laboratory, and histopathological parameters in patients undergoing limb-sparing surgery for primary malignant bone tumors. Favorable survival and functional outcomes were closely associated with factors such as high tumor necrosis rate, wide surgical margins, and absence of recurrence or metastasis. Laboratory markers like elevated LDH, ALP, and inflammatory indices proved valuable in risk stratification. Multivariate analysis confirmed that both tumor biology and surgical quality critically influence overall survival. These findings reinforce the importance of individualized, multidisciplinary approaches to optimize oncologic and functional results in limb-sparing procedures.

## Data Availability

The original contributions presented in the study are included in the article/supplementary material. Further inquiries can be directed to the corresponding author.
